# Purification and kinetics of the PHB depolymerase of *Microbacterium paraoxydans* RZS6 isolated from a dumping yard

**DOI:** 10.1371/journal.pone.0212324

**Published:** 2019-06-18

**Authors:** R. Z. Sayyed, S. J. Wani, Abdullah A. Alyousef, Abdulaziz Alqasim, Asad Syed, Hesham Ali El-Enshasy

**Affiliations:** 1 Department of Microbiology, PSGVP Mandal’s, Arts, Science, and Commerce College, Shahada Maharashtra, India; 2 Microbiology Research Group, Department of Clinical Laboratory Sciences, College of Applied Medical Sciences, King Saud University, Riyadh, Saudi Arabia; 3 Department of Botany and Microbiology, College of Science, King Saud University, Riyadh, Saudi Arabia; 4 School of Chemical and Energy Engineering, Faculty of Engineering, Universiti Teknologi Malaysia (UTM), Skudai, Johor Bahru, Malaysia; 5 City of Scientific Research and Technology Applications, New Burg Al Arab, Alexandria, Egypt; North Eastern Regional Institute of Science and Technology, INDIA

## Abstract

Poly-β-hydroxybutyrate (PHB) depolymerase is known to decompose PHB, biodegradable polymers and therefore has great commercial significance in the bioplastic sector. However, reports on PHB depolymerases from isolates obtained from plastic-contaminated sites that reflect the potential of the source organism is scarce. In this study, we evaluated the production of extracellular PHB depolymerase from *Microbacterium paraoxydans* RZS6 isolated from the plastic-contaminated site in the municipal area of Shahada, Maharashtra, India, for the first time. The isolate was identified using 16S rRNA gene sequencing, gas chromatographic analysis of fatty acid methyl esters (GC-FAME), and BIOLOG method. Ithydrolyzed PHB on minimal salt medium (MSM) containing PHB as the only source of carbon. The isolate produced PHB depolymerase at 45°C during 48 h of incubation. The enzyme was purified most efficiently using octyl-sepharose CL*-*4B column, with the highest purification yield of 6.675 Umg^-1^mL^-1^. The activity of the enzyme was enhanced in the presence of Ca^2+^ and Mg^2+^ ions but inhibited by Fe^2+^ (1 mM) ions and mercaptoethanol (1000 rpm). the nzyme kinetic analysis revealed that the enzyme was a metalloenzyme; requiring Mg^2+^ ions, that showed optimum enzyme activity at 30°C (mesophilic) and under neutrophilic (pH 7) conditions. Scale-up from the shake-flask level to a laboratory-scale bioreactor further enhanced the enzyme yield by 0.809 UmL^-1^. The molecular weight of the enzyme (40 kDa), as estimated by sodium dodecyl sulfate-polyacrylamide gel electrophoresis, closely resembled the PHB depolymerase of *Aureobacterium saperdae*. Our findings highlighted the applicability of *M*. *paraoxydans* as a producer of extracellular PHB depolymerase having potential of degrading PHB under diverse conditions.

## Introduction

Poly-β-hydroxy alkanoate (PHA) and poly-β-hydroxybutyrate (PHB) are stored as food and energy reserve in bacteria under the carbon-rich environment and are catabolized during nutrient stress conditions under the influence of PHB depolymerase [1–3]. PHB is a biocompatible, thermoplastic, nontoxic, completely biodegradable molecule exhibiting the properties of synthetic plastics. Moreover, it is easily degraded by PHB depolymerases and hence is an eco-friendly alternative to recalcitrant synthetic plastics [4–6]. PHB is degraded under natural conditions by the actions of PHB depolymerases produced by a wide variety of microorganisms [7–8]. Although PHB has commercial applications, identification of potent PHB degraders from relevant habitats and their evaluation for PHB depolymerase production, purification, and enzyme kinetic and scale-up must be carried out. Reports on bacterial PHB depolymerases isolated from plastic-contaminated sites, which reflect the true biodegradation potential of the enzyme, are scarce. Organisms isolated from plastic-contaminated sites that are capable of degrading PHB may serve as potential sources of efficient PHB depolymerases. Accordingly, in this study, we aimed to isolate PHB-degrading bacteria from plastic-rich dumping yards. We describe the isolation and polyphasic identification of a PHB depolymerase-producing *Microbacterium paraoxydans* RZS6 isolated from plastic-contaminated sites, production of PHB depolymerase, purification, characterization, and enzyme kinetics and scale-up of the identified PHB depolymerase.

## Materials and methods

### Chemicals and glassware

All the chemicals used in this study were of analytical research grade. PHB was purchased from Sigma-Aldrich (Germany); all other chemicals were purchased from Hi-Media Laboratories (Mumbai, India). The glassware was cleaned using 6N HCl and K_2_Cr_2_O_7_, rinsed with double-distilled water and dried in hot air oven.

### Isolation and screening of PHB depolymerase-producing bacteria

Sample collection, isolation, and screening of the PHB depolymerase-producing bacteria were performed as described by Wani et al. [4]. *M*. *paraoxydans* RZS6 was isolated from plastic-contaminated site located at latitude 21° 30′ 47.09″ N and longitude 74° 28′ 40.47″ E. This sampling site was purposefully chosen due to the higher probability of finding microflora that would be metabolically very active in biopolymer degradation.

### Selection of potent isolate

Isolate producing the zone of PHB hydrolysis were grown on MSM containing different concentrations of PHB (0.1–0.4%) at 30°C for 10 days [9]. The degradation of PHB was detected by observing the time profile of the growth of the isolate, and by observing the formation of the zone of PHB clearance surrounding the colonies. The level of PHB degradation was measured from the diameter of the zone of PHB hydrolysis.

### Temperature profile of the potent isolate

In order to assess the thermostability of the PHB depolymerase, the isolate was subjected to PHB degradation assays for a period of 10 days at 28°C, 37°C, and 45°C in MSM containing different concentrations (0.1%, 0.2%, 0.3%, and 0.4%) of PHB. The influence of temperature on PHB degradation was assessed by measuring the zone of PHB hydrolysis on each plate.

### Polyphasic identification of the isolate

Isolates showing the highest potential for PHB degradation on the PHB-agar were considered potent PHB depolymerase producers and were subjected to polyphasic identification.

#### Preliminary identification

Colonies of PHB-degrading isolate on nutrient agar (NA) medium were characterized using the Gram-staining method, morphological characteristic, and taxonomic characterization by biochemical kits (Hi-Media, Mumbai, India). The isolate was identified according to Bergey’s Manual of Determinative Bacteriology [10].

#### 16S rRNA gene sequencing

Sequencing of 16S rRNA genes of the isolate RZS6 was performed as per the method of Gangurde et al. [11]. DNA of the isolate was extracted according to the methods of Sambrook and Russel [12] using HiPurA Plant Genomic DNA Miniprep purification spin kit. Amplification of the 16S rRNA genes was performed using the following primers [13].

27f (5′-AGAGTTTGATCCTGGCTCAG-3′) and

1492r (3′-ACGGCTACCTTGTTACGACTT-5′).

The amplified sequences were analyzed by using gapped BLASTn (http://www.ncbi.nlm.nih.gov) search algorithm and the phylogenetic analysis was performed by evolutionary distance and maximum likelihood with 1,000 bootstrap replicates using the neighbor-joining method in Clustal W software [14]. The evolutionary distances were computed using the neighbor-joining method and the Kimura 2-parameter model. Phylogenetic trees were constructed according to the methods described by Tamura and Kumar [15]. The 16S rRNA gene sequences of the isolate were submitted to GenBank.

#### Fatty acid methyl ester (FAME) analysis

Fatty acids from whole cells of the isolate RZS6 were derivatized to methyl esters and analyzed by gas chromatography using the Sherlock Microbial Identification System (MIDI, Inc., Newark, DE, USA) [16]. Identification and quantification of fatty acids was carried out by comparing the retention time and peak area of the samples with those of standard fatty acids. Differences in the fatty acid profiles were computed using the Sherlock bacterial fatty acid ITSA1 aerobe reference library [17].

#### Phenotypic fingerprinting

Phenotypic fingerprinting of isolate RZS6 was carried out using a GEN III MicroPlate test panel with 95 carbon source utilization assays and a Microbial Identification System, 1998 (Biolog Inc., CA, USA) with Micro Log version 4.2 database software (Biolog Microstation System; Biolog Inc.) [18].

### Production and activity assay of PHB depolymerase

#### Growth kinetics and PHB depolymerase activity

Growth curve experiments were performed to evaluate the ability of the isolate to mineralize the substrate (PHB), as described by Maria and Zauscher [19]. The growth rate of *M*. *paraoxydans* RZS6 in PHB MSM was monitored over time at 620 nm in the presence of PHB as a substrate by withdrawing sample after every 12 h. The PHB depolymerase activity of the isolate was estimated as described by Papaneophytou et al. [7].

#### Production of PHB depolymerase

The production of PHB depolymerase was evaluated under shake-flask conditions by separately growing *M*. *paraoxydans* RZS6 at 30°C and 120 rpm for 4 days in MSM containing PHB (0.15% w/v) [20].

#### PHB depolymerase assay

After incubation, the MSM broth culture was centrifuged at 5000g for 15 min., and the PHB depolymerase activity of the supernatant was assayed as described by Papaneophytou et al. [7]. For this purpose, *M*. *paraoxydans* RZS6 (5 × 10^6^ cells mL^-1^) was grown in two reaction mixtures, each consisting of 50 mM Tris-HCl buffer (pH 7.0), 150 μgmL^-1^ PHB (prepared by sonication at 20 kHz for 15 min), and 0.5 mL of 2 mM CaCl_2_ at 30°C for 10 min. The PHB depolymerase activity was assayed as a decrease in the PHB turbidity at 650 nm [7]. One unit of PHB depolymerase activity was defined as the quantity of enzyme required to cause a 0.1 unit decrease in absorbance at 650 nm per min.

### Purification of PHB depolymerase

Purification of PHB depolymerase from cell-free supernatant was carried out by using three approaches as given below.

#### Ammonium sulfate precipitation

The crude PHB depolymerase in the supernatant was precipitated by the gradual addition of increasing concentrations (10–70% w/v) of ammonium salt. The precipitate so obtained was dialyzed overnight [8], followed by estimation of enzyme activity as per Papaneophytou et al. [7] and the protein concentration as per Lowry et al. method [21].,

#### Solvent purification method

The culture supernatant of the isolate was centrifuged at 5,000 g for 15 min, residues were dissolved in a pre-chilled 1:1 (v/v) mixture of acetone and ethanol and kept in a water bath at 50°C to allow the evaporation of the solvent. The pellet left over after solvent evaporation was dissolved in Tris-HCl buffer (pH 7), and the protein content and enzyme activity were assayed as described earlier [21,7].

#### Column chromatography

The culture supernatant of *M*. *paraoxydans* RZS6 was loaded on to octyl-sepharose CL*-*4B column charged with glycine*-*NaOH buffer (pH 9.0) and eluted using a 0–50% gradient of ethanol [22]. The fractions were collected and subjected to the estimation of protein content [21] and enzyme activity [7].

### Determination of molecular weight

The molecular weight of the purified PHB depolymerase of the isolate was determined using sodium dodecyl sulfate-polyacrylamide gel electrophoresis (SDS-PAGE) with standard molecular weight markers (GeNei^TM^, Bangaluru, India) such as phosphorylase B (82.2 kDa), bovine serum albumin (64.2 kDa), egg albumin (48.8 kDa), carbonic anhydrase (37.1 kDa), trypsin inhibitor (25.9 kDa), lysozyme (19.4 kDa), lysozyme (14.8 kDa), and lysozyme (6.0 kDa). Separated bands were stained for 4–6 h under shaking with 0.025% Coomassie brilliant blue G prepared in 40% (v/v) methanol and 7% (v/v) acetic acid followed by destaining with acetic acid: methanol (7:5) and 2X loading buffer (10 mL) and bands were observed under UV transilluminator. The protein concentration of the purified band was measured using bovine serum albumin as a standard [21].

### Enzyme kinetics

#### Effects of temperature on enzyme activity and determination of the thermostability of the enzyme

To determine the temperature optima and sensitivity of the PHB depolymerase, log culture of *M*. *paraoxydans* RZS6 (5×10^6^ cells mL^-1^) was grown in the reaction mixture at a different temperature ranging from 5 to 70°C for 10 min, and the enzyme activity was then measured as described above [7].

#### Effects of pH on enzyme activity and determination of the pH stability of the enzyme

The effect of pH on enzyme activity and the pH stability of the enzyme were determined in reaction mixtures having varying pH values in the range of 2 to 13. For this purpose log culture of *M*. *paraoxydans* RZS6 (5×10^6^ cells mL^-1^) was separately grown in reaction mixtures having different pH ranginging from 2 to 13 at 30°C for 10 min, and the enzyme activity was then measured as described above [7].

#### Effects of metal ions on the enzyme

In order to ascertain the metal requirement and type of metal required for the activity of the PHB depolymerase, *M*. *paraoxydans* RZS6 was separately grown in various reaction mixtures, each containing one type of metal ion, e.g., Ca^2+^, Mg^2+^, Mn^2+^, Cu^2+^, Co_2_^+^, Hg^2+^, Zn^2+^, and Fe^2+^ (1 mM), at 30°C for 10 min. Enzyme activity was then measured as described earlier [7].

#### Effects of solvents and chemicals on the enzyme

In order to determine the effects of solvents, such as methanol (10%, v/v), ethanol (10%, v/v), acetone (10%, v/v), mercaptoethanol (1%, v/v), tween*-*20 (1%, v/v), tween*-*80 (1%, v/v), ethylene diamine tetraacetic acid (EDTA; 1 mM), NaCl (1 mM), KCl (1 mM), and NaNO_3_ (1 mM), on the activity of the PHB depolymerase. The solvents and chemicals were added individually into each reaction mixture, followed by inoculation with the isolate RZS6, incubation at 30°C for 10 min, and measurement of enzyme activity [7].

### Scale up of the optimized process to a laboratory-scale bioreactor

In order to evaluate the performance of the organism in the bioreactor and to confirm the validity of the optimized shake-flask studies, the process was scaled-up to a fully-automated bioreactor of 5-L capacity (Model LF*-*5; Murhopye Scientific Co., Mysore, India). The bioreactor was sterilized, along with the above-optimized medium (working volume 3 L), at 121°C for 20 min; cooled, then inoculated with 3% (v/v) inoculum of *M*. *paraoxydans* RZS6 and incubated at 30°C for 48 h at 80 rpm. The samples were withdrawn after 12 h and subjected to estimation of protein concentrations [21] and enzyme activity [7].

### Statistical analysis

All the experiments were performed in triplicate and the mean of three replicates was considered. Each mean value was subjected to Student’s *t-*test and values of *P* ≤ 0.05 were taken as statistically significant [23].

## Results and discussion

### Isolation and screening of PHB depolymerase-producing bacteria

In total, 39 isolates were obtained from the respective plastic-contaminated sites; among these, seven isolates grew well and produced varying degrees of PHB hydrolysis on MSM containing PHB as the only carbon source. The isolate RZS6 produced the largest zone (27.9 mm) of PHB hydrolysis on MSM containing 0.4.% (w/v) of PHB and was therefore selected as the best PHB depolymerase producer. The hydrolysis of PHB reflected the ability of the isolate to produce PHB depolymerase.

Although several bacteria are known to secrete PHB depolymerase which degrades PHB, our findings demonstrated, for the first time, the production of PHB depolymerase from *M*. *paraoxydans* RZS6. The PHB depolymerase produced by isolate RZS6 obtained from the plastic-contaminated environment may be relevant for applications in plastic/bioplastic degradation. Mergaert et al. [24] have isolated 295 strains that degraded PHB and P (3HB-co*-*3HV) copolymer on MSM amended with PHB a sthe only carbon source. Elbanna et al. [25] reported *Schlegelella thermodepolymerans* and *Pseudomonas indica* K2 as PHA degraders on MSM. Additionally, Gangurde et al. [3] also reported PHB biodegradation by soil micro-flora on MSM containing PHB.

#### Selection of potent isolate

PHB-degrading isolate exhibited a range of PHB biodegradation abilities in MSM containing varying concentrations of PHB. Degradation of PHB was dependent on the amount of PHB in the medium. The degradation was significantly influenced by the concentration of the PHB in the medium. Maximum biodegradation was recorded at 0.2% concentration of PHB. However, among all PHB degrading isolates, RZS 6 showed significant degradation of PHB. In the case of other isolates, the zone diameter of PHB hydrolysis was comparatively smaller ranging from 0.9 to 2.9 mm. Variation in the degradation profile of PHB by different isolates suggested differences in their metabolic states [26]. Augusta et al. [27] have reported that the diffusion rate of an enzyme in plate assay differs with respect to the isolate and the amount of enzyme activity and rate of PHB degradation is influenced by environmental conditions. Kim et al. [28] also reported similar observations in the case of *Aspergillus* sp. strain NA*-*25.

### Temperature profile of the potent isolate

The isolate RZS6 exhibited optimum PHB degradation at 30°C with 0.2% PHB as a substrate. Importantly, the temperature profile of PHB degradation is dependent on the activity of the enzyme and therefore changes with the producing organism. Certain PHB depolymerases function in the mesophilic range of temperature, whereas others are thermotolerant or thermophilic in nature [6]. Wang et al. [29] have reported similar observations on a poly-depolymerase (3-hydroxybutyrate-co*-*3-hydroxy valerate) from *Acidovorax* sp. HB01.

### Polyphasic identification of potent PHB depolymerase producers

#### Preliminary identification

The phenotypic characteristics of the potent PHB-degrading isolate RZS6 were similar to those of *Microbacterium* sp.

#### 16S rRNA gene sequencing

**Fig 1:**
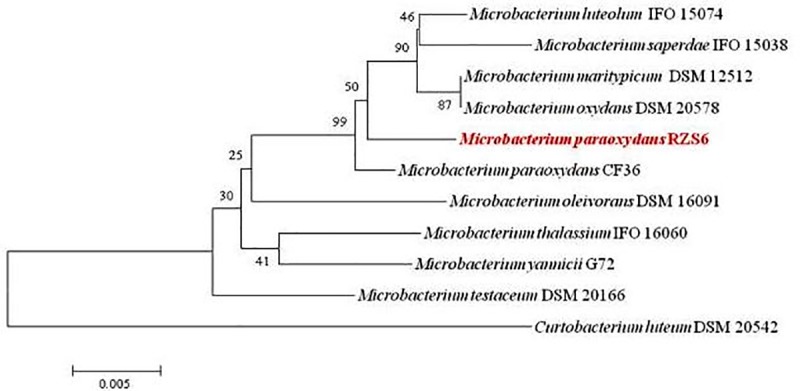
Phylogenetic analysis of *M*. *paraoxydans* RZS6 based on 16s rRNA gene sequence drawn using the neighbor joining method (MEGA 5.0 software) with evolutionary distances computed using Kimura’s two-parameter method showing the relationship of PHB depolymerase producing bacteria with the validly published sequences of related genera.

A BLAST search of the 16S rRNA gene sequences based on 1486 bp of the PHB depolymerase producing isolate with the 16S rRNA gene sequences of the NCBI GenBank database revealed the highest similarity and homology (99.2%) of the isolate RZS6 with *M*. *paraoxydans* (Fig 1). The phylogenetic tree based on 16S rRNA gene sequences of an isolate with the representative strains of its respective genus formed two distinct groups: Group I included the genus *Microbacterium* while Group II contained the strain *Curtobactrium leuteum* DSM 20542. Thus, based on phylogenetic analysis we identified the isolate RZS6 was identified as *M*. *paraoxydans*, The 16S rRNA gene sequence of the isolate was submitted to NCBI GenBank (http://www.ncbi.nlm.nib.gov/) under the name *M*. *paraoxydans* RZS6 with accession No. KP862607.

#### Whole-cell FAME analysis

The fatty acid profile of isolate RZS6 demonstrated the presence of characteristic fatty acids of *M*. *barkeri* (*Microbacterium barkeri*, *Corynebacterium*; similarity index: 0.859) and *M*. *chocolatum* (similarity index: 0.602) [17].

#### BIOLOG identification

The pattern of carbon source utilization assays for the isolate RZS6 demonstrated a maximum similarity index of 0.48 with *M*. *paraoxydans*.

Based on the preliminary characteristics, 16S rRNA gene sequencing, gas chromatographic analysis of FAMEs, and BIOLOG profiles, the isolate RZS6 was identified as *M*. *paraoxydans*. Based on 16s rRNA gene sequencing, GC-FAME analysis BIOLOG characteristics, the isolate RZS6 was identified as *Microbacterium paraoxydans* (Table 1).

**Table 1:** Identification of bacteria on the basis of 16S rRNA sequencing, GC FAME analysis, and BIOLOG profile.

Strain	16s rRNA sequencing	Base Pair	Identity (%)	GC FAME analysis	SI	BIOLOG identification	SI
RZS 6	*Microbacterium paraoxydans*	1486	99.2	*Microbacterium barkeri (Aureobacterium*, *Corynebacterium)*	0.859	*Microbacterium paraoxydans*	0.48

Phylogenetic analysis was carried out using the neighbor joining method. Evolutionary distances were computed using Kimura’s two-parameter method.

FaAME analyss was carried out by Sherlock Microbial Identification System and comparison with of bacterial fatty acid with standard ITSA1 aerobe reference library.

Phenotypic fingerprinting was carried out using a GEN III Micro Plate Microbial Identification System with Micro Log version 4.2 database software

### Production and assay of PHB depolymerase

#### Growth kinetics and PHB depolymerase activity

**Fig 2:**
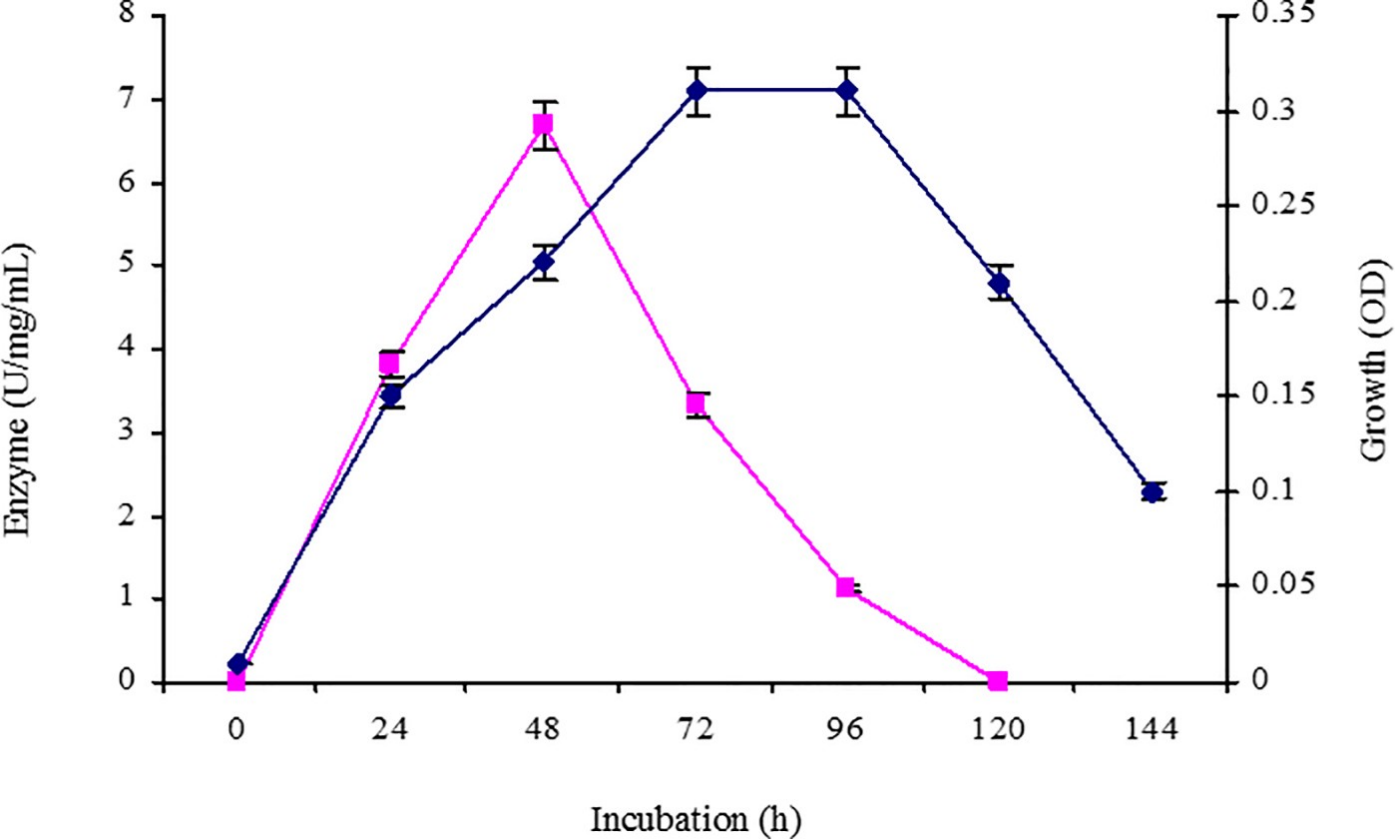
Growth kinetics and PHB depolymerase activity of *M*. *paraoxydans* RZS6 in MSM containing PHB as the only source of carbon, growth rate and enzyme activity were monitored over time at 620 and 50 nm respectively by withdrawing sthe ample after every 12 h.

Isolate RZS6 grew well in MSS containing PHB. The isolate showed faster growth, produced PHB depolymerase during the log phase of growth, and exhibited an optimum enzyme activity of 6.675 Umg^-1^mL^-1^, obtained at 48 h. The activity gradually decreased from the beginning of the stationary phase (72 h) and was completely absent during the decline phase (96–120 h) of growth (Fig 2).

#### Production of PHB depolymerase from the isolate

*M*. *paraoxydans* RZS6 produced copious amounts of PHB depolymerase in MSM. After 48 h (log phase) of incubation, RZS6 produced 6.675 U of PHB depolymerase with 0.247 mgmL^-1^ protein content in 2 days at 30°C. Gowda and Srividya [30] reported the production of 4 U of extracellular PHB depolymerase with a protein content of 0.05 mgmL^-1^ from *Penicillium expansum*. We report higher yields of PHB depolymerase.

### Purification of the PHB depolymerase

Having confirmed the presence of a PHB depolymerase in the cell-free supernatant of the MSM broth culture of *M*. *paraoxydans* RZS6, the supernatant was subjected to purification using three approaches, as described below.

#### Ammonium sulfate precipitation

**Fig 3:**
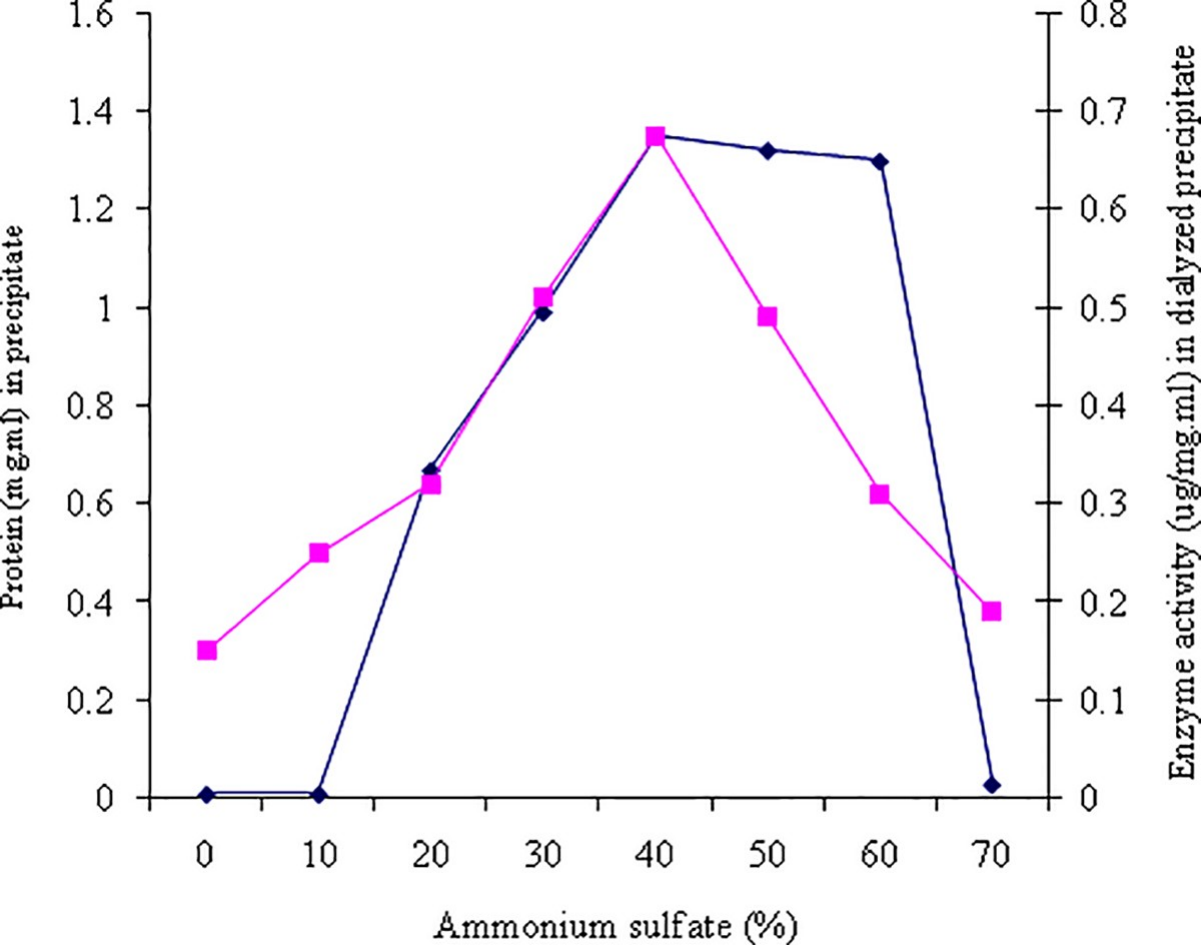
Ammonium sulfate purification of PHB depolymerase of *M*. *paraoxydans* RZS6. The enzyme was precipitated with increasing concentrations from 10–70% (w/v) of ammonium salt and the enzyme activity and and the protein were measured for each precipitation.

The maximum protein precipitation in the culture supernatant was obtained at 40% (v/w) concentration of ammonium sulfate. The protein concentrations, and enzyme activities in the dialyzed precipitate of *M*. *paraoxydans* RZS6 were 1.350 mgmL^-1^, and 6.6.75 U, respectively (Fig 3). Zhou et al. [31] also reported the precipitation of PHB depolymerase from *Escherichia coli* and *Penicillium* sp. DS9701-D2 using 70% and 75% ammonium sulfate. Shivakumar et al. [32] have reported efficient precipitation of PHB depolymerase from *Penicillium citrinum* S2 using 80% ammonium sulfate.

#### Solvent purification method

Solvent mixture of acetone and ethanol (1:1) adversely affected PHB depolymerase activity, leaving behind only 45.66% enzyme activity. This significant loss in enzyme activity may be related to the precipitation of proteins (enzymes) by acetone and ethanol. Thus, the solvent purification method proved to be inefficient.

#### Column chromatography

Out of the five fractions, fraction 1, 2 and 5 did not show any enzyme activity, fractions 4 was less in enzyme activity (1.013 U) and protein content (0.062 mgmL^-1^), fraction 3 exhibited the maximum enzyme activity of 7.703 U, with 0.247 mgmL^-1^ protein content. Other fractions. Thus, column chromatography using an octyl-sepharose CL*-*4B column resulted in the most efficient purification. Purification of PHB depolymerase from various organisms has been carried out using Sephadex columns [33–34]. Wang et al. [29], DEAE-Sepharose column and using column chromatography [6–8].

### Determination of the molecular weight of the purified enzyme

**Fig 4:**
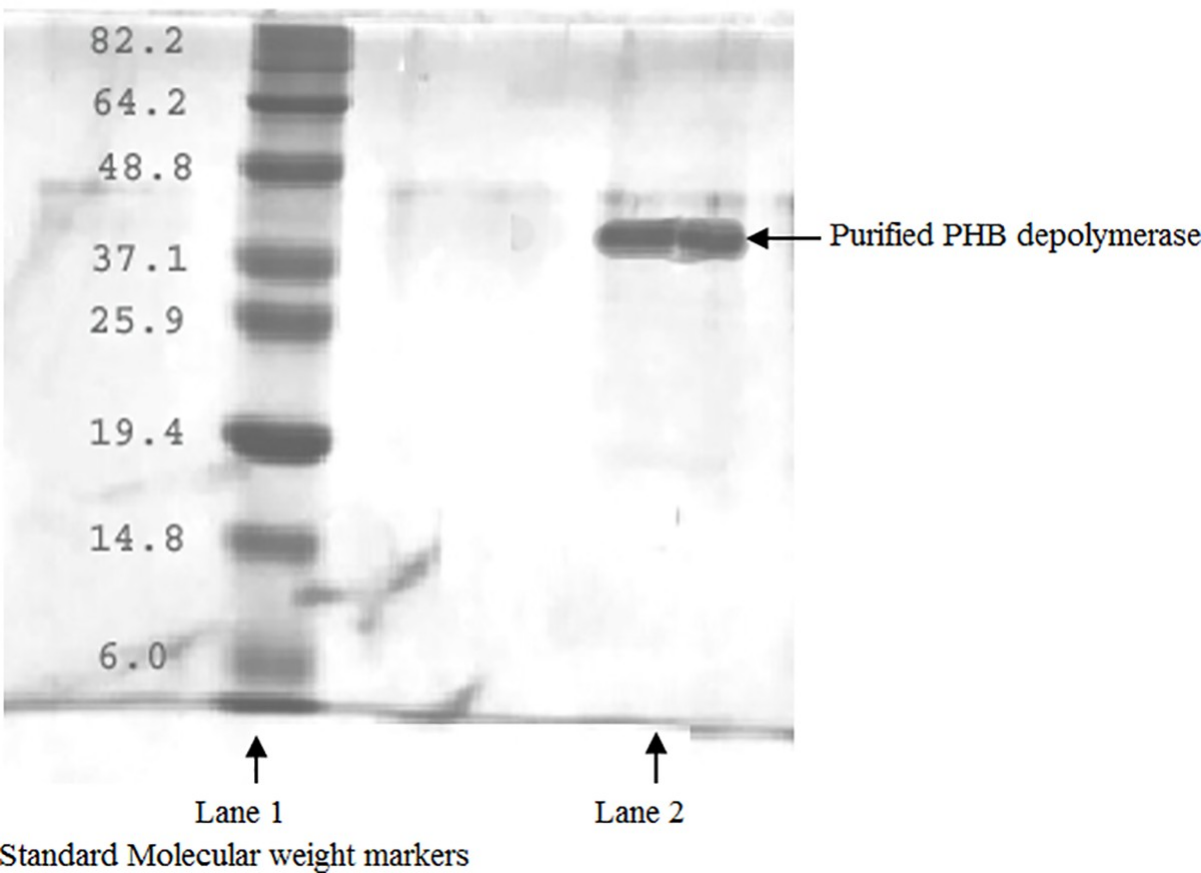
SDS-PAGE for determination of molecular weight of PHB depolymerase of *M*. *paraoxydans* RZS6 by comparing it with with standard molecular weight markers as shown in Lane 1 and seprated band of PHB depolymerase shown in Lane 2. Separated bands were stained with Coomassie brilliant blue G and destained with acetic acid:methanol (7:5) and observed under UV transilluminator.

The purified protein fraction from *M*. *paraoxydans* RZS6 yielded single protein bands corresponding to the molecular weight of approximately 40 kDa (Fig 4). Sadocco et al. [35] also reported a PHB depolymerase of 42.7 kDa from *Aureobacterium saperdae*. Calabia and Tokiwa [36] identified a PHB depolymerase of 41 kDa from *Streptomyces* sp. MG 41.

### Enzyme kinetics

#### Effects of temperature and determination of the thermostability of the enzyme

**Fig 5:**
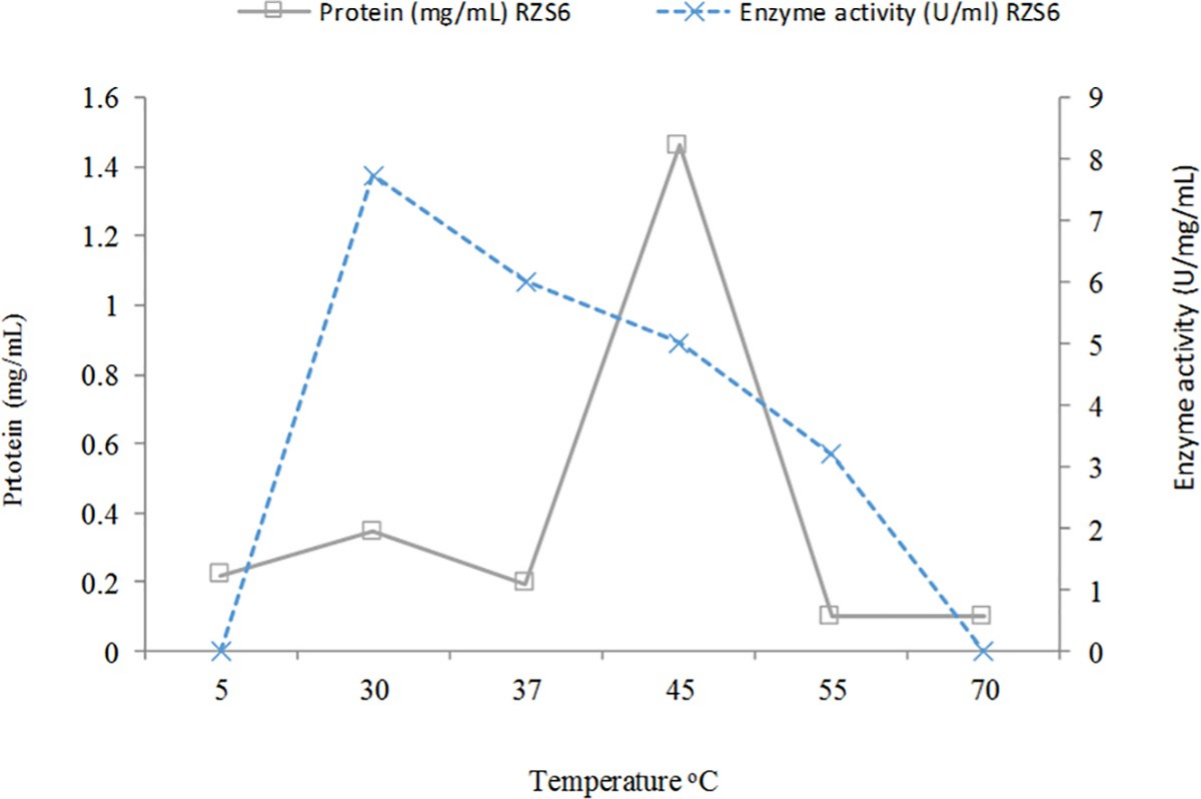
Effects of temperature and determination of thermostability of PHB depolymerase of *M*. *paraoxydans* RZS6. Enzyme activity was measured by separaley incubating the enzmyme in the reaction mixture at various temperature ranging from 5 to 70°C for 10 min.

The PHB depolymerase of RZS6 showed an optimum enzyme activity of 6.657 U with 0.247 mgmL^-1^ protein content at 30°C, indicating the mesophilic nature of the enzyme. The enzyme activity decreased with as the temperature increase, and the enzyme was completely inactivated at 70°C (Fig 5). The decrease in the enzyme activity with the increase in temperature reflected the thermolabile nature of the enzyme. Wang et al. [29] and Gowda and Shivakumar [30] reported thermostable PHB depolymerases in *Pseudomonas mendocina* DSWY0601 and *Penicillium expansum*, respectively. Calabia and Tokiwa [36] reported optimum PHB depolymerase activity in *Streptomyces* sp. MG at 50°C.

#### Effects of pH on enzyme activity and determination of the pH stability of the enzyme

**Fig 6:**
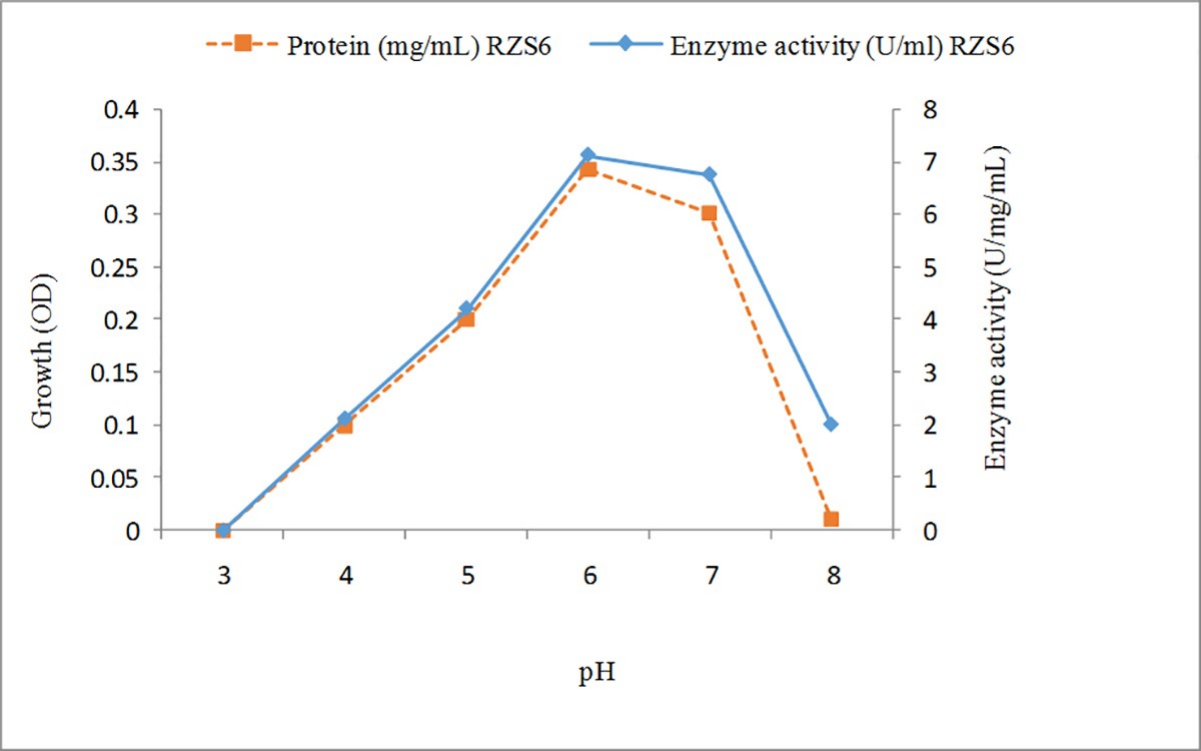
Effects of pH and determination of pH stability of PHB depolymerase of *M*. *paraoxydans* RZS6. Enzyme activity was measured by separaley incubating the enzmyme for 10 min in the reaction mixture of various pH ranging from 2–13.

The optimum activity of the PHB depolymerase of *M*. *paraoxydans* RZS6 was obtained at pH (Fig 6), indicating the neutrophilic nature of the enzyme. The enzymes from isolate RZS6 remained stable at an acidic pH (4.0–6.5). Sadocco et al. [35] and Jeong [37] reported an optimum pH for PHB depolymerase from *A*. *saperdae* in the range of 7.0 to 9.0. The pH sensitivity of the PHB depolymerases of *Penicillium expansum* and *Pseudomonas mendocina* DSWY0601 has been reported to be between 6.0 and 9.0 respectively [29–30]. Soam et al. [6] reported pH 7.0 as the optimum pH for enzyme production in *Bacillus mycoides*. Calabia and Tokiwa [36] reported that the optimum PHB depolymerase activity of *Streptomyces* sp. MG was in the pH range from 6.5 to 8.5.

#### Effects of metal ions on enzyme activity

The presence of Mg^2+^ ions significantly enhanced PHB depolymerases however, Fe^2+^ ions negatively affected the enzyme activity, leaving behind only 22.26% activity. The other metal ions had a negligible effect on the enzyme activity (Table 2). The increase in PHB depolymerase activity in the presence of Mg^2+^ ions was attributed to the enzyme activator nature of these metal ions. Wang et al. [29] also reported a positive effect of Mg^2+^ and Ca^2+^ ions on the PHB depolymerase of *Pseudomonas mendocina* DSWY0601.

**Table 2:** Effects of metal ions and chemicals on the PHB depolymerase of *M*. *paraoxydans* RZS6.

Metal ion (1 mM)	Enzyme activity (UmL^-1^)	Protein content (mgmL^-1^)	Specific activity (Umg^-1^mL^-1^)	% activity
No metal ions	0.146 (0.010)	0.247 (0.013)	0.6210 (0.022)	**59.10** (0.119)
Mg^2+^	0.194 (0.100)	0.247 (0.013)	0.7854 (0.031)	**78.54** (0.172)
Ca^2+^	0.192 (0.095)	0.247 (0.013)	0.7773 (0.030)	77.73 (0.161)
Mn^2+^	0.129 (0.081)	0.247 (0.013)	0.5627 (0.017)	56.27 (0.149)
Cu^2+^	0.099 (0.072)	0.247 (0.013)	0.4008 (0.016)	40.08 (0.132)
Co^2+^	0.101 (0.075)	0.247 (0.013)	0.4089 (0.016)	40.89 (0.136)
Hg^2+^	0.097 (0.086)	0.247 (0.013)	0.3927 (0.012)	39.27 (0.123)
Zn^2+^	0.094 (0.080)	0.247 (0.013)	0.3805 (0.012)	38.05 (0.125)
Fe^2+^	0.055 (0.050)	0.247 (0.013)	0.2226 (0.113)	22.26 (0.016)
**Chemicals**				
No chemical	0.134 (0.011)	0.219 (0.013)	0.6118 (0.019)	61.18 (0.014)
NaCl	0.154 (0.019)	0.219 (0.013)	0.7196 (0.022)	**71.96** (0.022)
NaNO_3_	0.148 (0.100)	0.219 (0.013)	0.6757 (0.031)	67.57 (0.031)
Methanol	0.145 (0.092)	0.219 (0.013)	0.6621 (0.019)	66.21 (0.019)
KCl	0.138 (0.073)	0.219 (0.013)	0.6301 (0.018)	63.01 (0.018)
Ethanol	0.112 (0.074)	0.219 (0.013)	0.5114 (0.014)	51.14 (0.014)
Tween-80	0.11 (0.088)	0.219 (0.013)	0.5022 (0.009)	50.22 (0.009)
Tween-20	0.103 (0.069)	0.219 (0.013)	0.4703 (0.008)	47.03 (0.008)
Acetone	0.091 (0.086)	0.219 (0.013)	0.4155 (0.007)	41.55 (0.007)
EDTA	0.046 (0.079)	0.219 (0.013)	0.2100 (0.005)	21.00 (0.005)
Mercaptoethanol	0.023 (0.051)	0.219 (0.013)	0.1050 (0.003)	10.50 (0.003)

Values were taken to be statistically significant at P ≤ 0.05

Values are the mean of three replicates

Values in parenthesis are the values generated by comparing control with the test by the Students *t-*test

#### Effects of different chemicals on enzyme activity

Mercaptoethanol caused the maximum inhibition (85%) of enzyme activity. NaCl enhanced the activity of the PHB depolymerase (Table 2). The loss of enzyme activity in the presence of chemicals and solvents occur due to denaturation and proteolysis of the enzyme. Papaneophytou et al. [7] have also reported mercaptoethanol as a strong inhibitor of PHB depolymerase in *Thermus thermophilus* HB8. Wang et al. [29] have also reported the negative effects of ethanol, acetone, tween, and other chemicals on the PHB depolymerase of *Pseudomonas mendocina* DSWY0601.

### Scale up of the optimized process to a laboratory-scale bioreactor

Scale-up of the parameters optimized at the shake-flask level to the laboratory-scale bioreactor level enhanced PHB depolymerase yield from 7.703 to 8.512 U and protein content from 0.247 to 0.297 mgmL^-1^. An increase of 0.809 UmL^-1^ in enzyme yield and 0.50 mgmL^-1^ in protein content was obtained.

## Conclusion

The occurrence of PHB-degrading bacteria in plastic-contaminated sites and their growth in the presence of PHB on MSM containing PHB as the only source of carbon indicated their ability to degrade bioplastic (PHB). The growth and maximum enzyme activity of isolate at themesophillic temperature indicated the ability of an organism to survive well under environmental conditions of dumping yard. The highest purification yield obtained by using octyl-sepharose CL*-*4B column reflects the binding and adsorption specificity of the enzyme to sepharose. Enhanced activitt of enzyme in the presence of metal ions such as Ca^2+^ and Mg^2+^ indicated the role of these metal ions as activators of the enzyme and metalloenzyme nature of the PHB depolymerase. Thus, PHB depolymerase-producing bacteria obtained from plastic-contaminated environments are expected to be important catalyst for applications in the biodegradation of bioplastics. However, further studies on the growth of *Microbacterium paraoxydans* and performance of PHB depolymerase under natural conditions shall help in this direction.
